# Angiogenesis in nasopharyngeal carcinoma: insights, imaging, and therapeutic strategies

**DOI:** 10.3389/fonc.2024.1331064

**Published:** 2024-05-28

**Authors:** Chenxi Xia, Jia Zhao, Yu Huang, Hongbin Miao, Feipeng Zhao

**Affiliations:** ^1^ Department of Otolaryngology-Head and Neck Surgery, the Affiliated Hospital of Southwest Medical University, Southwest Medical University, Luzhou, Sichuan, China; ^2^ Department of Otolaryngology-Head and Neck Surgery, Chengdu Second People’s Hospital, Chengdu, Sichuan, China; ^3^ Department of Otolaryngology-Head and Neck Surgery, Bishan hospital of Chongqing Medical University, Bishan Hospital of Chongqing, Bishan, Chongqing, China

**Keywords:** nasopharyngeal carcinoma, angiogenesis, Epstein–Barr virus, exosomes, anti-angiogenic therapy

## Abstract

Nasopharyngeal carcinoma (NPC) is a highly prevalent head and neck malignancy in southern China frequently diagnosed at advanced stages owing to subtle early symptoms and associated metastasis. Angiogenesis emerges as a pivotal factor in NPC progression, with numerous angiogenesis-related factors showing aberrant expression and contributing to increased neovascularization within NPC tumors. These abnormal vessels not only nourish tumor growth but also facilitate metastasis, culminating in unfavorable patient outcomes. Multiple studies have demonstrated the applicability of various imaging techniques for assessing angiogenesis in NPC tumors, thus serving as a foundation for personalized treatment strategies and prognostic assessments. Anti-angiogenic therapies have exhibited significant potential for inhibiting NPC angiogenesis and exerting anti-tumor effects. To enhance efficacy, anti-angiogenic drugs are frequently combined with other treatment modalities to synergistically enhance anti-tumor effects while mitigating the side effects associated with single-agent therapies, consequently improving patient prognosis. Identifying the potential mechanisms and key targets underlying NPC angiogenesis and exploring more effective detection and treatment approaches holds promise for shaping the future of NPC diagnosis, treatment, and prognosis, thereby offering new avenues and perspectives for research and clinical practice.

## 1 Introduction

Nasopharyngeal carcinoma (NPC) is a prevalent head and neck cancer in Southeast Asia, particularly in southern China, and often presents challenges due to its hidden location and nonspecific early symptoms (1, 2). As an epithelial carcinoma originating from the nasopharyngeal mucosa, NPC exhibits high sensitivity to radiotherapy (3). However, its propensity for local recurrence and distant metastasis often leads to treatment failure and poor prognosis (4–6). The precise mechanism underlying distant metastasis of NPC remains elusive; nevertheless, there is a consensus that angiogenesis significantly contributes to this process.

NPC represents a pathological ecosystem wherein a close relationship exists between the tumor and the host, with the vascular system serving as an integral component (7). Angiogenesis is an essential step in tumor progression, which not only facilitates the supply of nutrients crucial for tumor growth but also serves as a conduit for eliminating the metabolic waste generated by cells. In contrast, tumor cells release pro-angiogenic factors that stimulate the proliferation and migration of vascular endothelial cells, ultimately leading to the formation of new blood vessels. This intricate process accelerates tumor growth and facilitates metastasis (8). Moreover, newly formed blood vessels within the tumor exhibit immaturity and high permeability, resulting in inadequate perfusion of the tumor and the creation of a hypoxic microenvironment (9). This further promotes tumor invasion and metastasis while inhibiting the effectiveness of immune cells and reducing the diffusion and efficacy of chemotherapy drugs. Clinically, these factors contribute to poor prognosis (10).

Currently, radiotherapy is widely considered the primary treatment for early-stage non-metastatic NPC. However, when early distant metastasis occurs, radiotherapy combined with chemotherapy is more effective in improving the prognosis (1, 2, 11). However, resistance to chemoradiotherapy results in unsatisfactory therapeutic effects (4–6). NPC angiogenesis plays a crucial role in tumor progression (12), significantly affecting both patient outcomes and prognosis, highlighting its considerable clinical value and prospects for anti-angiogenic therapy. Therefore, gaining a comprehensive understanding of the research advancements and applications of angiogenesis-related NPC is of tremendous importance for treating advanced or metastatic NPC.

## 2 Transcriptional regulation of angiogenesis in NPC

### 2.1 VEGF

As a highly specific mitogen of endothelial cells, vascular endothelial growth factor (VEGF) can strongly stimulate endothelial cell proliferation to induce angiogenesis and lymphangiogenesis (13). When VEGF expression levels increase in the tumor tissues or serum of NPC patients, it typically closely correlates with TNM staging, leads to a higher risk of distant metastasis, and lowers long-term survival rates; therefore, it could be a potential marker for distant metastasis and prognosis in NPC (14–18). VEGF serves as a prognostic indicator for NPC patients and is positively associated with an unfavorable prognosis (15, 17). Serum VEGF detection proves beneficial in predicting tumor metastasis and the clinical outcome of the patients (14, 16). VEGF expression is significantly correlated with microvessel density (MVD), which is a potent indicator of vasculogenesis (19). VEGF and MVD in NPC show a positive correlation with tumor progression and stage (20). Secreted by NPC cells VEGF promotes tumor angiogenesis, cell invasion, and migration (21). Exosomes released by tumor cells can stimulate VEGF-related pathways, fostering angiogenesis (22, 23). EBV-encoded proteins can contribute to promoting tumor growth and progression by interacting with VEGF (24, 25). VEGF is subject to regulation by various factors. For instance, the pro-angiogenic effect of chemotaxis-related factors is mediated through the regulation of VEGF expression (26). In the hypoxic conditions resulting from extensive tumor growth, activated HIF-1α stimulates VEGF transcription (27). In the angiogenesis mechanism, VEGF can activate the Ras/MAPK and PI3K/AKT pathways by influencing matrix metalloproteinases and cyclooxygenase-2 in NPC (21, 28). Conversely, when VEGF function is disrupted, angiogenesis is prevented in NPC (29). Clinically, quercetin, a plant extract, along with several tyrosine kinase inhibitors, inhibits angiogenesis by targeting VEGF or its receptors (29–32). Gene polymorphisms, specifically VEGF-460T/C and VEGF-2578C/A, can influence the risk and invasiveness of NPC. These variations are significant in guiding the assessment of clinical outcomes in patients, potentially associated with angiogenesis (33, 34).

### 2.2 ANG

Angiogenin (ANG) is a secreted ribonuclease with proangiogenic properties. Angiogenin-2 (ANG-2) plays a beneficial role in promoting angiogenesis in NPC (35). Particularly, ANG-2 has been reported to exert an anti-vascular effect in NPC, which is highly dependent on VEGF expression levels. When endogenous VEGF is absent, ANG-2 overexpression decreases the tumor microvascular density, thereby playing an antitumor role (36). Furthermore, angiotensin- (1–7) [ANG- (1–7)] can against the tumor angiogenic process by reducing the expression of VEGF and hypoxia-inducible factor-1α (HIF-1α) (37).

### 2.3 HIF-1α

HIF-1α is an important regulatory factor in the adaptation of tumor cells to anoxic environmental conditions (27). It can serve as an indicator of hypoxia within tumors and is intricately linked to processes such as angiogenesis, invasion, metastasis, energy metabolism, and resistance to tumor radiotherapy. During the NPC angiogenic process, HIF-1α is regulated by many factors. To achieve Epstein–Barr virus (EBV)-induced vasculogenic mimicry (VM), EBV stimulates the expression of HIF-1α, which is also activated by latent membrane protein 2A (LMP2A) (38). Additionally, EBV-miR-BART1-5P (39), NPC-extracellular vesicle (EV) -derived miR-144 (40), chemokine (C-C motif) ligand (CCL5) (41), EBV- Epstein-Barr nuclear antigen 1 (EBNA1) (25), forkhead box M1 (42), BART10-5p and miR-18a (43) can increase HIF-1α synthesis to bring about tumor angiogenesis and progression. NPC tissue growth leads to the formation of a hypoxic microenvironment. In this hypoxic condition, HIF-1α expression can be inhibited by α-momorcharin, causing the NPC vessel formation suppressed (44).

### 2.4 MMPs

Matrix metalloproteinases (MMPs) are a group of endogenous peptidases that degrade extracellular matrix (45). Exosomal MMP-13 plays a pro-angiogenic role in NPC (46). MMPs also act as mediators of other factors that enhance angiogenesis in NPC. MMP-9 cooperates with the protease-activated receptor 2 and EBV-encoded latent membrane protein 1 (LMP1) to induce vasculogenesis in NPC (47). Ras-like estrogen-regulated growth inhibitor (RERG) can suppress MMPs’ expression and influence tumor vessel formation (48). In another study, RBMS3 (RNA binding motif, single-stranded interacting protein 3) indirectly inhibits the angiogenic effects of MMP-9 and MMP-2 (49). ADAMTS9, a member of the ADAMTS (a disintegrin-like and metalloproteinase (reprolysin type) with thrombospondin type 1 motif) metalloproteinase family, downregulates MMP-9 and VEGF-A expression to inhibit NPC growth and vessel formation (50).

### 2.5 NF-кB

Nuclear transcription factor кB (NF-кB), a family of transcription factors, is involved in the regulation of tumor angiogenesis and growth. According to some studies, NF-κB can restrain the ability to promote NPC neovascularization by tumor suppressor genes, such as transforming growth factor-β binding protein 2 (51), RERG (48), cylindromatosis lysine 63 deubiquitinase (52) and NFKB inhibitor beta (53). Additionally, it has been reported that upregulated receptor-interacting serine/threonine kinase 4 (RIPK4) (54), LMP1 (55, 56), and pregnancy upregulated nonubiquitous calmodulin (CaM) kinase (PNCK) (57) are related to the activation of NF-кB signaling pathway, which results in tumor vessel formation and disease progression.

## 3 EBV-associated NPC angiogenesis factors

**Figure 1:**
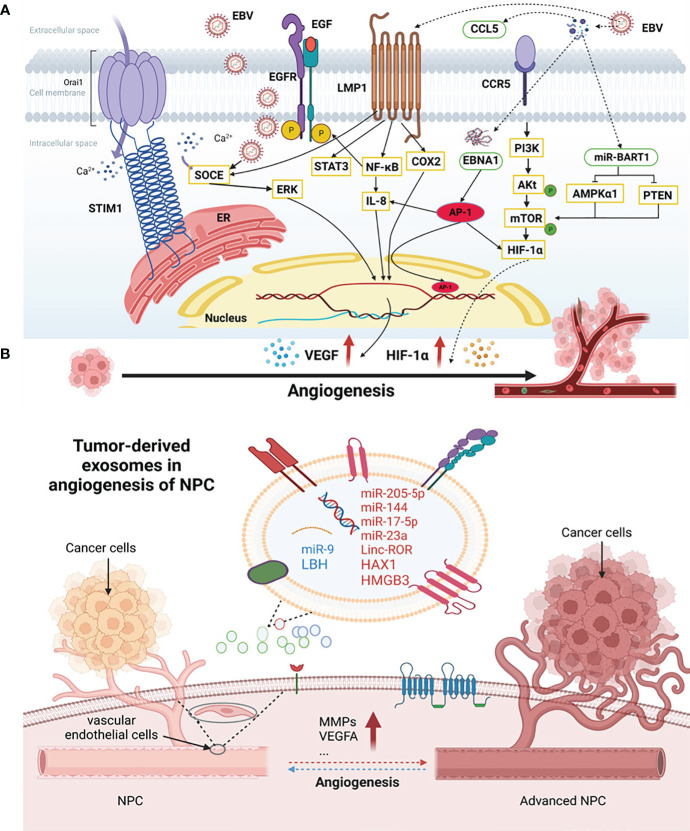
The association of EBV infection and exosomes with angiogenesis in NPC. **(A)** EBV-associated NPC angiogenesis factors. EBV and some genes encoded by EBV could activate the angiogenesis in NPC. EGF, epidermal growth factors; EGFR, epidermal growth factor receptors; SOCE, store-operated Ca2+ entry; STIM1, stromal interaction molecule 1; LMP1, latent membrane protein 1; STAT3, signal transducer and activator of transcription 3; IL-8, interleukin-8; COX-2, cyclooxygenase-2; EBNA1, Epstein-Barr nuclear antigen 1; AMPKα1, AMP-activated protein kinase; PTEN, tensin homolog deleted on chromosome ten; CCL5, chemokine (C-C motif) ligand; CCR5, C-C chemokine receptor type 5; ERK, extracellular regulated kinase; PI3K, phosphoinositide 3-kinase; mTOR, mammalian target of the rapamycin; VEGF, vascular endothelial growth factor; HIF-1α, hypoxia-inducible factor-1α; ER, endoplasmic reticulum. **(B)** Tumor-derived exosomes in angiogenesis of NPC. NPC cells secreted exosomes which can be internalized and absorbed by endothelial cells and tumor cells themselves, promoting the angiogenic process. HAX1, HS1-related protein X-1; HMGB3, high-mobility group box 3; LBH, limb-bud and heart; VEGF, vascular endothelial growth factor; MMPs, matrix metalloproteinases.

In addition to host genetics, EBV infection may be the most common causal agent of NPC (2) (**Figure 1A**). The positive correlation between plasma EBV DNA load and NPC risk not only makes it a valuable tool for NPC screening (58) but also underscores its close association with NPC neovascularization. EBV could activate stromal interaction molecule 1 (STIM1) -dependent Ca^2+^ signaling to promote vasculogenesis in NPC (59) and increased expression of the angiogenic factor CCL5, promoting NPC vessel formation through modulation of the PI3K/AKT and HIF-1α pathways (41). In NPC, EBV could promote VM by activating the LMP2A-mediated PI3K/AKT/mTOR/HIF-1α signaling pathway (38).

Moreover, some genes encoded by EBV actively participate in NPC neovascularization (**Figure 1A**). LMP1, a proto-oncogene encoded by EBV, induces angiogenesis through several signaling pathways (60). It promotes the expression of epidermal growth factor receptors (EGFR) in NPC cells by activating the NF-κB pathway and signal transducer and activator of transcription 3 (STAT3), which affects angiogenesis and enhances EBV infection in NPC cells (55). LMP1 also promotes VM through the VEGF/VEGFR1 signaling pathway (61). Under the influence of extracellular epidermal growth factors (EGF), LMP1 enhances VEGF-mediated angiogenesis by facilitating store-operated Ca^2+^ entry (SOCE) (24). Additionally, LMP1 can induce VEGF expression through the JAK/STAT and MAPK/ERK pathways to upregulate tumor vasculogenesis (62). In the NPC cell line NPC-KT, LMP1 induces interleukin-8 (IL-8) expression to regulate angiogenesis by activating the NF-κB pathway (56). Research shows that LMP1-induced cyclooxygenase-2 (COX-2) may play a role in NPC angiogenesis (63).

Moreover, EBV-encoded EBNA1 stimulates the expression of transcription factor AP-1, which can promote NPC neovasculogenesis by targeting IL-8, VEGF, and HIF-1α (25). EBV-encoded RNAs promote angiogenesis by stimulating vascular cell adhesion molecule-1 expression (64). EBV-miR-Bart1-5p, a key miRNA encoded by EBV, can directly target AMP-activated protein kinase (AMPKα1) and activate the AMPK/mTOR/HIF-1 pathway, inducing abnormal glycolysis and angiogenesis in NPC cells (39).

## 4 Exosome-associated NPC angiogenesis factors

Exosomes, a subtype of EVs, are crucial mediators of intercellular communication, being vesicular structures secreted by cells and containing proteins, nucleic acids, and other bioactive molecules that facilitate this process. Exosomes secreted by tumor cells can be internalized and absorbed by endothelial cells and tumor cells themselves, promoting angiogenesis and a premetastatic niche, ultimately leading to tumor progression (65) (**Figure 1B**). Similarly, exosomes in NPC have been reported to be associated with pathological angiogenesis, distant metastasis, resistance to chemoradiotherapy, and immunosuppression, and their influence extends to the tumor microenvironment (66).

lncRNAs, miRNAs, and other non-coding RNAs are important components of exosomes derived from tumor cells and participate in NPC neovascularization. Linc-ROR, a long-stranded exosomal non-coding RNA of nasopharyngeal origin, promotes NPC proliferation, migration, and angiogenesis via the p-AKT/p-VEGFR2 pathway (67). Exosome miR-205-5p plays a vasostimulatory role in NPC by activating the EGFR/ERK signaling pathway and MMPs expression by targeting desmocollin-2 (68). MiR-144 in EVs derived from NPC plays a role in promoting tumor progression through the FBXW7/HIF-1a/VEGF-A axis, which promotes angiogenesis (40). MiR-17-5p, which is highly expressed in NPC, targets bone morphogenetic protein and activin membrane-bound inhibitor and regulates the AKT/VEGF-A signaling pathway to increase angiogenesis (22). MiR-23a plays an important role in NPC neovasculogenesis by acting on TSGA10 (69). However, exosomal miR-9 secreted by NPC cells inhibits vascular formation and metastasis by targeting midkine and regulating PDK/AKT signaling (70).

Other factors derived from NPC exosomes have been reported. HS1-related protein X-1 (HAX1) in EVs enhances the expression level of integrin β6 and regulates the FAK pathway to promote tumor angiogenesis (71, 72). High-mobility group box 3 (HMGB3) of nuclear exosome origin accelerates pathological vasculogenesis in NPC (73). NPC exosomes containing limb-bud and heart (LBH) inhibit epithelial-mesenchymal transformation and angiogenesis by regulating VEGF-A (74). Additionally, PFKFB-3 (Enzymes 6-phosphofructo-2-kinase/fructose-2, 6-bisphosphatase-3) (75), MMP13 (46), intercellular adhesion molecule-1 (ICAM-1), CD44 variant isoform 5 and platelet response protein-1 (76) in exosomes are involved in the regulation of NPC angiogenesis.

## 5 Other NPC angiogenesis factors

There are many studies on NPC angiogenesis. For instance, the proangiogenic lncRNA LINC00240 functions by inhibiting the expression of miR-26a-5p (77). The enhancer of zeste homolog 2 (EZH2), which is highly expressed in NPC, can activate NPC neovasculogenesis via the EZH2-miR-1-ET-1 axis and promote the proliferation, migration, and VM of NPC cells (78). Additionally, kinesin family member 2A (79), vimentin (80), tripartite motif-containing 24 (81), CD93 (82), TWIST (12), and annexin A2 (83) exert angiogenic effects on NPC. Instead, it is reported that in NPC human METCAM/MUC18 (84), inhibitor of growth 4 (ING4), PTEN (phosphatase and tensin homolog deleted on chromosome ten) (85), IkappaB kinase alpha (86), PTPRG (protein tyrosine phosphatase, receptor type G) (87), fibulin-2 (88) and latent TGF-β binding protein 2 (89) plays an anti-angiogenic role. In addition to the aforementioned factors, hypoxia can trigger angiogenesis of NPC (90).

## 6 Angiogenic application in NPC

### 6.1 Angiogenesis-related imaging studies in NPC

Abnormal microangiogenesis can aggravate hypoxia, which is an important factor affecting treatment resistance and poor prognosis of NPC. Dynamic contrast-enhanced magnetic resonance imaging (DCE-MRI) is a noninvasive technique that reflects capillary permeability, angiogenic activity, tumor angiogenesis, blood perfusion, and hypoxic status of NPC tissues. Remarkably, some parameters in DCE-MRI are associated with positive expression of HIF-1α in NPC, which can provide the basis for the formulation of individualized treatment for NPC patients (91). Due to significant changes in dynamic parameters during radiotherapy, DCE-MRI can monitor NPC angiogenesis during treatment and quantitatively evaluate the effects of tumor treatment (60). Multiple studies have shown that magnetic resonance perfusion-weighted imaging (MR-PWI) (92) and diffusion-weighted imaging (DWI) also reflect NPC angiogenesis and radiotherapy sensitivity. Common parameters in DWI assessments include pure molecular diffusion (D) and perfusion-related diffusion (D*). Research has indicated that elevated D* values correlate with increased angiogenesis and parenchymal perfusion in NPC (93, 94). The D value was significantly decreased in primary NPC; however, elevated D and D* values indicated radiosensitivity of the cancer, suggesting a favorable prognosis for the tumor.

Contrast-enhanced ultrasound (CEUS) is an imaging technique that assesses tumor vasculogenesis by examining the diffusion patterns of contrast agents within a tumor. It has been claimed that MVD in NPC grafts can be reflected by CEUS parameters in a nude mouse model (95). Additionally, dynamic contrast-enhanced ultrasonography (DCE-US) can be used to evaluate the efficacy of anti-angiogenic therapy in NPC (96).

### 6.2 Angiogenesis-related therapy in NPC

In recent years, various new treatment schemes have been developed, among which anti-angiogenesis therapy has emerged and has been used in clinical practice to inhibit the malignant progression of tumors by blocking or inhibiting the related regulatory pathways (**Table 1**) (**Supplementary Table 1**).

**Table 1:** Anti-angiogenesis drugs in nasopharyngeal carcinoma.

Drug	Model system	Reported Regulatory pathway	Ref.
**Bevacizumab**	clinic application	VEGF↓	(97–100)
**Endostar**	clinic application; mice	VEGF↓; Disrupting the hypoxic environment	(101–106)
**Tyrosine kinase inhibitors:** apatinib, famitinib, sunitinib, lenvatinib	clinic application	Blocking the binding of VEGF and VEGFR	(29, 31, 32, 107–109)
**Morphine**	cell line;mice	Unclear	(110)
**Valsartan and losartan**	clinic application; mice	VEGF-A↓, ANG-2↓	(111)
**Traditional Chinese medicines:** Rhizoma Curcumae, quercetin, triptolide, traditional herbal formula NPC01	cell line;mice	VEGF↓; NF-κB↓	(30, 112–114)

VEGF, vascular endothelial growth factor; ANG-2, angiogenin-2; NF-кB, nuclear transcription factor кB. The meaning of symbol “↓” is “blocking” or “inhibiting”.

Bevacizumab, a monoclonal antibody targeting VEGF, effectively inhibits VEGF activity, thereby achieving the objectives of anti-tumor angiogenesis and metastasis suppression in NPC (97). This therapy can be used for the treatment of locally advanced and metastatic NPC (98–100). Recent studies have demonstrated that Endostar, a recombinant human endostatin, is a targeted drug with antiangiogenic and antitumor effects, and when combined with other antitumor therapies, it significantly enhances the overall anticancer effect (101, 102). Endostar significantly enhances radiosensitivity in NPC, reducing the side effects of radiotherapy. Moreover, it can be combined with chemotherapy or chemoradiotherapy to improve the prognosis of patients with metastatic NPC (101, 103, 104). The effect of endostar is related to the downregulation of VEGF expression (105) and could correct the pathological angiogenesis process to disrupt the hypoxic environment in tumor tissues (106).

Many kinase inhibitors can also be employed as anti-angiogenic drugs for NPC treatment. Apatinib, famitinib, sunitinib, and lenvatinib are tyrosine kinase inhibitors that inhibit MVD in patients with NPC. The mechanism of tumor angiogenesis inhibition by apatinib is related to blocking the binding of VEGF and VEGFR. Studies have confirmed that apatinib can achieve good efficacy in the treatment of NPC patients with lung metastasis and advanced VEGFR-2-negative NPC while ensuring safety (107). Apatinib can be used not only as a monotherapy but also in combination with radiotherapy or chemotherapy to treat patients with NPC, effectively enhancing its anti-vascular effects (29, 108). Famitinib combined with radiotherapy increased the radiosensitivity of NPC cells by inhibiting angiogenesis (109). As a single agent, sunitinib significantly inhibited tumor growth and angiogenesis in NPC xenografts. These effects can be enhanced when combined with chemotherapy (32). For anti-angiogenic drug-resistant NPC, lenvatinib has been shown to effectively reverse the resistance of NPC with high FGF-2 expression (31). Additionally, the angiokinase inhibitor BIBF 1120 has an antitumor angiogenic effect and can be used in combination with cisplatin to treat NPC (115).

Many traditional Chinese medicines and their extracts have antitumor effects in the antivascular treatment of NPC. Bioinformatics analysis showed that Rhizoma Curcumae could inhibit angiogenesis in NPC (112). After treatment with quercetin, VEGF expression and NF-κB activity were decreased, and endothelial cell tube formation was inhibited (30). Li Yanwei et al. found that NPC01, an ancient recipe from the Song Dynasty of China, may play an anti-angiogenic role in cancer by inhibiting the effects of pro-angiogenic factors HIF-1α and VEGF (113). Triptolide, a traditional Chinese medicinal extract combined with radiotherapy, inhibits the growth and angiogenesis of NPC (114).

Furthermore, some drugs have unexpected effects in anti-angiogenic therapy for NPC, in addition to their traditional effects. Low doses of the opioid analgesic morphine in NPC led to chemoresistance, which was surprisingly associated with reduced tumor neovascularization, whereas high doses had the opposite effect (110). Angiotensin II receptor blockers (ARBs), which are commonly used in cardiovascular diseases, not only have traditional antihypertensive effects but also have anti-angiogenic effects in NPC by promoting cell apoptosis (111).

## 7 Conclusions

Angiogenesis is an essential process for the distant metastasis and local recurrence of NPC; however, the underlying mechanism is intricate and ambiguous. Based on the current state of researches, key factors such as VEGF, ANG, HIF-1α, MMPs, and NF-κB play a pivotal role in the angiogenesis of NPC (**Supplementary Table 2**). Recent findings have highlighted the regulatory roles of EBV infection and exosomes in angiogenesis. In clinical practice, DCE-MRI and CEUS have proven to be effective imaging techniques for angiogenesis detection. Furthermore, the use of antiangiogenic drugs, such as bevacizumab and Endostar, either alone or in combination with other chemotherapy drugs, has demonstrated significant potential for enhancing the prognosis of patients with NPC.

The ongoing advancements in angiogenesis research within NPC offer a novel avenue for the identification of biomarkers. The miRNAs within exosomes and the distinctive molecules linked to EBV imply a potential correlation with the angiogenesis and metastasis of NPC. Subsequent investigations are warranted to identify more precise and clinically significant markers related to exosomes and EBV. These may offer valuable insights for clinical diagnosis and prognosis of NPC.

Multi-targeted anti-angiogenic approach may emerge as a more efficacious strategy for NPC treatment. However, the reported clinical anti-angiogenesis drugs are limited, and their targets exhibit relative simplicity. EBV participates in NPC angiogenesis through diverse pathways. Exploring crucial targets and developing drugs targeting Epstein-Barr virus-mediated angiogenesis could represent a novel avenue for anti-angiogenic interventions. Combining such approaches with anti-angiogenic drugs may enhance the efficacy of NPC treatment in the future.

In summary, a better understanding of angiogenesis provides new insights into the mechanism of NPC. Advancements in key factors, EBV infection, and exosomes hold promise for enhancing NPC diagnosis, treatment, and prognosis, paving the way for future research and clinical application of NPC.

## Author contributions

CX: Conceptualization, Writing – original draft, Writing – review & editing. JZ: Conceptualization, Investigation, Writing – original draft. YH: Methodology, Writing – original draft. HM: Methodology, Writing – original draft. FZ: Funding acquisition, Supervision, Writing – review & editing.
