# Supplementary material for: Angiogenesis in nasopharyngeal carcinoma: insights, imaging, and therapeutic strategies
Source: Front Oncol. 2024 May 28;14:1331064. doi: 10.3389/fonc.2024.1331064 (PMC11165036; doi:10.3389/fonc.2024.1331064)
Supplement: Supplementary file 1 [file Table_1.docx]

**Supplementary Table 1. Clinical trials of anti-angiogenesis drugs in NPC.**

| **Clinical trial no.** | **NPC type** | **Antiangiogenesis drugs used** | **Clinical trial phase** | **Number of patients enrolled(n)** | **Concentration of antiangiogenic drug** | **PFS (%)** | **OS** | **Ref.** |
| --- | --- | --- | --- | --- | --- | --- | --- | --- |
| 1 | Metastatic NPC | Endostar | II | 28 | Endostar 0.03 mg/ml/day | (1-year)69.8 | (1-year)90.2% | (101) |
| 2 | Locally advanced NPC | Endostar | Not recorded | 25 | Endostar 7.5 mg/m2/day | (5-years)68.0 | (5-years)76.0% | (102) |
| 3 | Recurrent or metastatic NPC | Bevacizumab | II | 39 | Bevacizumab 7.5 mg/kg | (6-months)79.5 | 21.0 months (median OS) | (100) |
| 4 | NPC with hypertension | ARBs | Not recorded | 548 | Not recorded | Not recorded | (5-years)87.8% | (111) |

PFS, progression-free survival rate; OS, overall survival; ARBs, Ang II receptor blockers.
