# Supplementary material for: Angiogenesis in nasopharyngeal carcinoma: insights, imaging, and therapeutic strategies
Source: Front Oncol. 2024 May 28;14:1331064. doi: 10.3389/fonc.2024.1331064 (PMC11165036; doi:10.3389/fonc.2024.1331064)
Supplement: Supplementary file 2 [file Table_2.docx]

**Supplementary Table 2. Major transcriptional regulation of angiogenesis in NPC.**

| **Angiogenesis factors** | **Molecular functions** | **Reference** |
| --- | --- | --- |
| **VEGF** | It can strongly stimulate endothelial cell proliferation, induce angiogenesis and lymphangiogenesis, which is related to invasion and metastasis of the cancer. | (13-19, 21) |
| **ANG** | ANG-2 acts as an inhibitor of angiogenesis by affecting the connection between endothelial cells and perivascular cells, but acts as a synergistic promoter when VEGF is present. ANG-(1-7) can inhibit NPC angiogenesis by decreasing the expression of VEGF and HIF-1α. | (35-37) |
| **HIF-1α** | It activates the downstream hypoxic response genes under hypoxic conditions, thereby increasing angiogenesis and swelling to promote the proliferation and migration of cancer cells. | (27) |
| **MMPs** | It activates the angiogenic process by degrading type IV collagen in the basement membrane and releasing angiogenic factors bound to the ECM. | (45, 46) |
| **NF-кB** | It regulates angiogenesis by acting on endogenous VEGF. | (30, 57, 114) |

VEGF, vascular endothelial growth factor; ANG, angiogenin; HIF-1α, hypoxia-inducible factor-1α; MMPs, matrix metalloproteinases; NF-кB, nuclear transcription factor кB; ECM, extracellular matrix.
